# Association between anxiety and aggression in adolescents: a cross-sectional study

**DOI:** 10.1186/s12887-019-1479-6

**Published:** 2019-04-18

**Authors:** Jee Eun Chung, Gonjin Song, Kitai Kim, Jeong Yee, Joo Hee Kim, Kyung Eun Lee, Hye Sun Gwak

**Affiliations:** 1 College of Pharmacy, Institute of Pharmaceutical Science and Technology, Hanyang University, 55 Hanyangdaehak-ro, Sangnok-gu, Ansan, 15588 South Korea; 2 College of Pharmacy and Division of Life and Pharmaceutical Sciences, Ewha Womans University, 52 Ewhayeodae-gil, Seodaemun-Gu, Seoul, 03760 South Korea; 3 Department of Communication, Honam University, 417 Eodeung-daero, Gwangsan-gu, Gwangju, 62399 South Korea; 4 College of Pharmacy, Ajou University, 206 Worldcup-ro, Yeongtong-gu, Suwon, 16499 South Korea; 5 College of Pharmacy, Chungbuk National University, 660-1 Yeonje-ri, Osong-eup, Heungdeok-gu, Cheongju, 28160 South Korea

**Keywords:** Anxiety, Aggression, Adolescent, Revised Children’s manifest anxiety scale, The aggression questionnaire

## Abstract

**Background:**

The purpose of this study was to investigate the relationship between anxiety proneness and aggressive behavior in adolescents.

**Methods:**

A quantitative, large scale cross-sectional study was conducted in Korea. The survey questionnaire included general health behavior and scales for assessing anxiety (Revised Children’s Manifest Anxiety Scale; RCMAS) and aggressive behavior (The Aggression Questionnaire; AQ) in adolescents.

**Results:**

A total of 2432 students participated in the survey, and 1933 individuals completed the questionnaire, indicating a response rate of 79.5%. Based on RCMAS, 163 (8.4%) subjects were classified as the anxiety group. Aggressive behavior was significantly associated with higher anxiety scores. In particular, among four subdomains of aggression, anger and hostility had a stronger relationship with anxiety than did physical and verbal aggression. Multivariate analysis demonstrated that anxiety was independently associated with gender, age, headache, constipation, asthma, and aggression score. Adolescents with total aggression scores of 69 or higher showed a 9-fold (AOR = 9.00, CI = 6.33–13.51) higher risk of anxiety compared to those with under 69.

**Conclusion:**

Aggression and anxiety are important aspects of mental health in adolescents. Our results demonstrated that higher risk of anxiety was associated with total aggression scores. In particular, indirect aggression (i.e. anger and hostility) was more closely associated with anxiety than direct aggression.

## Introduction

Adolescence is a critical developmental period by which social, emotional, and physical changes to the body can build up negative self-perceptions [1]. Previous studies have shown that adolescent behavior is highly determined by emotions [2, 3], whereas aggression in adolescent males was a risk factor for the development of internalizing problems such as anxiety and depression [4]. In addition, there is growing concern for the co-occurrence of behavioral, emotional and cognitive problems.

Cumulative prevalence by age 16 is estimated to be 9.9% for adolescents meeting the diagnostic criteria for anxiety and 23% for behavioral disorders in the U.S [5]. Behavioral disorders often involve aggressive behavior that can be manifested physically, verbally, and socially. Aggression is the most widely researched of all child behavior problems and is described in two main forms, namely direct-physical aggression and indirect-relational aggression, depending on their method of harm [6]. Direct aggression harms others by damaging their physical well-being and includes physically and verbally aggressive behavior, while indirect aggression harms others by damaging social relationships [7].

While aggression and anxiety have been studied separately as two distinct properties, researchers have begun to suspect that anxiety may be one of the key emotional underpinnings of childhood aggression and their link is not unidirectional. Several studies have reported the link between anxiety and aggression in childhood. It was revealed that reactively aggressive children at age six were significantly more anxious than their non-aggressive counterparts [8]. Also, in elementary school students, relational and physical aggression were suggested as the strongest predictors of anxiety [9]; in addition, a study on American 2nd, 3rd, and 4th graders demonstrated a relationship between baseline anxiety symptoms and higher levels of relational aggression over a 1-year period [10]. Furthermore, a study including children with attention-deficit/hyperactivity disorder reported that disruptive behavioral disorders were associated with reactive–proactive aggression and anxiety sensitivity [11]. While these studies provide insight regarding childhood aggression and anxiety, they focus on relatively young children (in their elementary school years or younger), making it difficult to apply them to adolescents.

Adolescence is a unique period in human development, with rapid physiological and psychological changes. Due to these changes, adolescents often face a number of crises and dilemmas, especially in the areas of mental and emotional health. Kim et al. revealed that Korean middle and high-school students experience high level of stress related to general studying demands and preparations for college admission that may lead to serious physical or psychological problems [12]. Researchers have reported that aggressive adolescent behavior is associated with academic pressure. These exam pressures could lead to the generation of negative emotional symptoms within students [13].

Adolescent stress has been linked to negative mental health outcomes such as anxiety and depression [14]. Anxiety may be interpreted as an emotional response of an aversive situation, and several studies were conducted to investigate family and school environment factors associated with anxiety in Korean adolescents [15–17]. Depression and anxiety are the strongest predictors of suicidal ideation, threats, and plans [18]. The increase in internalizing distress throughout adolescence is particularly concerning given that suicide has been the leading cause of death among Korean youths aged 15–19 [19]. Thus, developing a more in-depth understanding of the relationship between anxiety and aggression throughout adolescence is of paramount importance. Despite this significance, few studies have linked anxiety and aggression in adolescents.

Therefore, the purpose of this study was to examine the associations among subdomains of aggression and anxiety disorders and investigate associated factors with anxiety disorders among Korean adolescents.

## Methods

### Study population

A cross-sectional study was conducted with randomly selected students from middle schools (7th–9th grade) and high schools (10th–12th grade) in Gwangju, South Korea, April–May 2016. With the assistance of statisticians at the Office of Education of the region, six clusters in Gwangju city were formed based on the socio-demographic characteristics of each cluster. In addition, the questionnaire was distributed during researchers’ on-site visit to schools. Each participant voluntarily completed the survey. Once completed, unique study identification was assigned to each participant to ensure confidentiality and anonymity. A total of 2432 students participated in the survey, and 1933 individuals completed the questionnaire, showing a response rate of 79.5%. All procedures performed in studies involving human participants were in accordance with the ethical standards of the institutional and/or national research committee and with the 1964 Helsinki declaration and its later amendments or comparable ethical standards.

### Measurements

The survey was comprised of the following questionnaires: Revised Children’s Manifest Anxiety Scale (RCMAS) [20, 21] and Aggression Questionnaire (AQ) [22]. Symptoms of anxiety were measured using the Korean version of RCMAS originally developed by Reynolds and Richmond [21]. It is a self-reported screening tool (Cronbach’s α = 0.94) to measure anxiety in children and adolescents age 6–19. The RCMAS consists of 37 items, each requiring a yes or no answer. Three anxiety subscales are included: physiological anxiety, worry/oversensitivity, and social concerns. A total score of 25 or greater is clinically significant and children with a total score of 34 or above were referred to a psychiatric clinic for further assessment. In this study, participants were classified as anxiety group if their total score was 25 or higher. The Korean version of AQ was used to assess the levels of aggression among participants [22]. The AQ was developed by Buss and Perry and was translated and revised into Korean (Cronbach’s α = 0.86). The scale consists of 29 items scored on a 5-point Likert scale. This scale contains four subdomains: (a) physical aggression, (b) verbal aggression, (c) anger, and (d) hostility. The total possible scores range from 29 to 145, with higher scores denoting higher level of aggression.

Demographic information included age, gender, caffeine intake, alcohol consumption, smoking, history of medical symptoms such as headache, muscle pain, scoliosis, constipation, indigestion, heartburn, atopic dermatitis, sinusitis and asthma, and medication history of consuming painkillers, digestants and sleeping pills within 30 days.

### Statistical analysis

The independent t-test was used to compare continuous variables between participants with and without anxiety or aggression propensity. The chi-square test was used for categorical variables, and data were expressed as percentages. A multivariate analysis of variance (MANOVA) was conducted to examine differences in the subdomains of aggression propensity (physical aggression, verbal aggression, anger, hostility, and total), since each of the subdomains of aggression was correlated with at least one other subdomain. To analyze the relationship between anxiety and aggression propensity, Pearson’s correlation coefficient was used. The area under the receiver operator characteristics (AUROC) curve was calculated for the cut-off of aggression scores. Multivariable logistic regression analysis was performed, using the backward stepwise method. The anxiety and control groups were classified according to the RCMAS (control group: RCMAS < 25, anxiety group: RCMAS ≥25). Odds ratio (OR) and adjusted OR (AOR) were calculated with 95% confidence interval (CI). The model fit of the prediction model was assessed by an analysis of the AUROC. *P* value of less than 0.05 was considered statistically significant. Statistical analysis was conducted using SPSS Statistics for Windows 20.0 (IBM Cop., Armonk, NY).

## Results

The mean age was 15.0 ± 1.9 years and 897 (47.1%) were boys. The distribution of the students across the schools was as follows: 930 students (48.1%) in middle school, 1001 (51.8%) in high school, and 2 (0.1%) unspecified. Among them, a total of 163 (8.4%) adolescents were classified as anxiety group based on RCMAS, and 69.9% of them were girls. Adolescents under the age of 15 were more likely to be in anxiety group than those older than 15. As shown in Table 1, participants in anxiety group consumed more caffeine, had headaches, myalgia, scoliosis, constipation, indigestion, heartburn, and asthma than did those in the non-anxiety group. The medication history of painkillers, digestants and sleeping pills was significantly associated with anxiety proneness. However, smoking status and alcohol consumption have failed to reach the statistical significance.

**Table 1:** Demographics of participants

	Control group (%)	Anxiety group (%)	*p*-value
Sex			< 0.001
Boys	848 (48.8)	49 (30.1)	
Girls	891 (51.2)	114 (69.9)	
Age, years			0.008
≤ 15	1002 (58.0)	112 (68.7)	
> 15	725 (42.0)	51 (31.3)	
Smoking			0.489
Yes	86 (5.0)	6 (3.8)	
No	1640 (95.0)	154 (96.3)	
Alcohol			0.161
Yes	327 (18.9)	38 (23.5)	
No	1402 (81.1)	124 (76.5)	
Caffeine			0.045
Yes	1435 (84.0)	144 (90.0)	
No	273 (16.0)	16 (10.0)	
Self-reported medical conditions			
Headache			< 0.001
Yes	256 (15.2)	66 (40.5)	
No	1483 (84.8)	97(59.5)	
Muscle pain			< 0.001
Yes	495 (28.5)	81 (49.7)	
No	1244 (71.5)	82 (50.3)	
Scoliosis			0.002
Yes	113 (6.5)	21 (12.9)	
No	1626 (93.5)	142 (87.1)	
Constipation			< 0.001
Yes	134 (7.7)	26 (16.0)	
No	1605 (92.3)	137 (84.0)	
Indigestion			< 0.001
Yes	188 (10.8)	39 (23.9)	
No	1551 (89.2)	124 (76.1)	
Heartburn			< 0.001
Yes	89 (5.1)	21 (12.9)	
No	1650 (94.9)	142 (87.1)	
Atopic dermatitis			0.785
Yes	253 (14.6)	25 (15.3)	
No	1486 (85.5)	138 (84.7)	
Sinusitis			0.079
Yes	38 (2.2)	21 (12.9)	
No	1701 (97.8)	142 (87.1)	
Asthma			0.009
Yes	38 (2.2)	9 (5.5)	
No	1701 (97.8)	154 (94.5)	
Medication			
Pain reliever			< 0.001
Yes	374 (21.5)	57 (35.0)	
No	1365 (78.5)	106 (65.0)	
digestant			0.011
Yes	185 (10.6)	28 (17.2)	
No	1554 (89.4)	135 (82.8)	
Sleeping pill			0.031
Yes	19 (1.1)	5 (3.1)	
No	1719 (98.9)	158 (96.9)	

Control group: RCMAS ≤25, Anxiety group: RCMAS > 25

As described in Table 2, baseline characteristics were compared for each subdomain of aggression such as physical, verbal aggression, anger and hostility. Overall, young age and the male sex were risk factors of aggression propensity. Higher scores of aggression were revealed in adolescents with caffeine and alcohol consumption, smoking, complaints of headaches, myalgia, scoliosis, constipation, indigestion, heartburn, and sinusitis. The propensity for aggressive behavior was associated with consuming pain relievers, digestants and sleeping pills.

**Table 2:** Univariate analysis of factors associated with aggressive propensity

	Physical	Verbal	Anger	Hostility	Total	p-
	45	25	35	40	145	value
Sex						< 0.001
Boys	18.8 ± 5.6^***^	11.1 ± 3.8^***^	15.4 ± 5.0^*^	16.6 ± 6.5	61.9 ± 16.9^**^	
Girls	16.5 ± 5.1	10.4 ± 3.6	15.9 ± 4.9	17.0 ± 6.6	59.8 ± 16.3	
Age, years						< 0.001
≤ 15	17.6 ± 5.4	10.5 ± 3.7^*^	15.8 ± 4.8	16.9 ± 6.6	60.9 ± 16.5	
> 15	17.5 ± 5.6	11.1 ± 3.8	15.4 ± 5.1	16.7 ± 6.5	60.7 ± 16.8	
Smoking						< 0.001
Yes	21.5 ± 6.1^***^	11.9 ± 3.9^**^	17.0 ± 5.0^**^	17.6 ± 6.3	68.1 ± 16.6^***^	
No	17.4 ± 5.4	10.7 ± 3.7	15.6 ± 4.9	16.8 ± 6.6	60.5 ± 16.6	
Alcohol						< 0.001
Yes	19.2 ± 5.5^***^	11.5 ± 3.8^***^	16.9 ± 5.5^***^	17.9 ± 6.7^*^	65.5 ± 17.1^***^	
No	17.2 ± 5.4	10.6 ± 3.7	15.4 ± 4.8	16.6 ± 6.5	59.7 ± 16.3	
Caffeine						0.001
Yes	17.6 ± 5.5	10.8 ± 3.7	15.9 ± 4.9^***^	17.1 ± 6.6^***^	61.4 ± 16.5^*^	
No	17.0 ± 5.6	10.5 ± 4.0	14.6 ± 4.9	15.6 ± 6.5	57.7 ± 16.9	
Self-reported medical conditions						
Headache						< 0.001
Yes	18.6 ± 6.0^***^	11.5 ± 4.0^***^	17.2 ± 5.5^***^	19.4 ± 7.7^***^	66.6 ± 18.9^***^	
No	17.3 ± 5.4	10.6 ± 3.7	15.4 ± 4.8	16.3 ± 6.2	59.6 ± 15.9	
Muscle pain						< 0.001
Yes	18.2 ± 5.8^*^	11.4 ± 3.8^***^	16.8 ± 5.3^***^	18.5 ± 6.9^***^	64.8 ± 17.6^***^	
No	17.3 ± 5.3	10.5 ± 3.6	15.2 ± 4.7	16.1 ± 6.3	59.1 ± 15.9	
Scoliosis						0.005
Yes	18.2 ± 6.1	11.2 ± 3.8	15.9 ± 5.6	18.6 ± 7.5^*^	63.9 ± 19.2^*^	
No	17.5 ± 5.4	10.7 ± 3.7	15.7 ± 4.9	16.7 ± 6.8	60.6 ± 16.4	
Constipation						< 0.001
Yes	17.7 ± 6.2	11.0 ± 3.9	17.2 ± 5.5^***^	19.1 ± 7.2^***^	64.9 ± 18.4^*^	
No	17.5 ± 5.4	10.7 ± 3.7	15.5 ± 4.9	16.6 ± 6.5	60.4 ± 16.4	
Indigestion						< 0.001
Yes	17.4 ± 5.7	10.8 ± 3.7	16.9 ± 5.5^***^	19.3 ± 7.3^***^	64.3 ± 17.8^*^	
No	17.6 ± 5.5	10.7 ± 3.7	15.5 ± 4.8	16.5 ± 6.4	60.4 ± 16.4	
Heartburn						0.001
Yes	18.4 ± 6.3	11.4 ± 3.8	17.5 ± 5.2^***^	19.2 ± 7.5^***^	66.5 ± 18.7^***^	
No	17.5 ± 5.4	10.7 ± 3.7	15.6 ± 4.9	16.7 ± 6.5	60.5 ± 16.4	
Atopic dermatitis						0.093
Yes	18.2 ± 5.7^*^	10.8 ± 3.8	16.2 ± 5.1	17.5 ± 6.9	62.8 ± 17.7^*^	
No	17.4 ± 5.4	10.7 ± 3.7	15.6 ± 4.9	16.7 ± 6.5	60.5 ± 16.4	
Sinusitis						0.023
Yes	18.9 ± 6.1^*^	11.4 ± 3.7^*^	16.5 ± 4.9^*^	17.7 ± 6.3	64.5 ± 16.6^*^	
No	17.4 ± 5.4	10.7 ± 3.7	15.6 ± 4.9	16.8 ± 6.6	60.5 ± 16.6	
Asthma						0.678
Yes	18.2 ± 5.5	10.5 ± 3.8	16.2 ± 5.1	17.6 ± 7.1	62.5 ± 17.6	
No	17.5 ± 5.5	10.8 ± 3.7	15.7 ± 4.9	16.8 ± 6.6	60.8 ± 16.6	
Medication						
Pain reliever						< 0.001
Yes	18.0 ± 5.8	11.3 ± 3.9^***^	16.8 ± 5.3^***^	18.0 ± 7.0^***^	64.1 ± 17.7^***^	
No	17.4 ± 5.4	10.6 ± 3.7	15.4 ± 4.8	16.5 ± 6.4	59.9 ± 16.2	
Digestants						0.002
Yes	18.3 ± 5.8^*^	11.5 ± 3.8^*^	16.6 ± 5.0^*^	18.5 ± 6.7^***^	65.0 ± 16.8^***^	
No	17.5 ± 5.4	10.6 ± 3.7	15.6 ± 4.9	16.6 ± 6.5	60.3 ± 16.5	
Sleeping pill						0.002
Yes	21.2 ± 7.7^*^	12.9 ± 4.8^*^	17.0 ± 6.5	19.8 ± 9.0^*^	70.9 ± 23.2^*^	
No	17.5 ± 5.4	10.7 ± 3.7	15.7 ± 4.9	16.8 ± 6.5	60.7 ± 16.4	

^*^p < 0.05, ^**^p < 0.01, ^***^p < 0.001

Data were expressed as the mean ± S.D

**Fig. 1:**
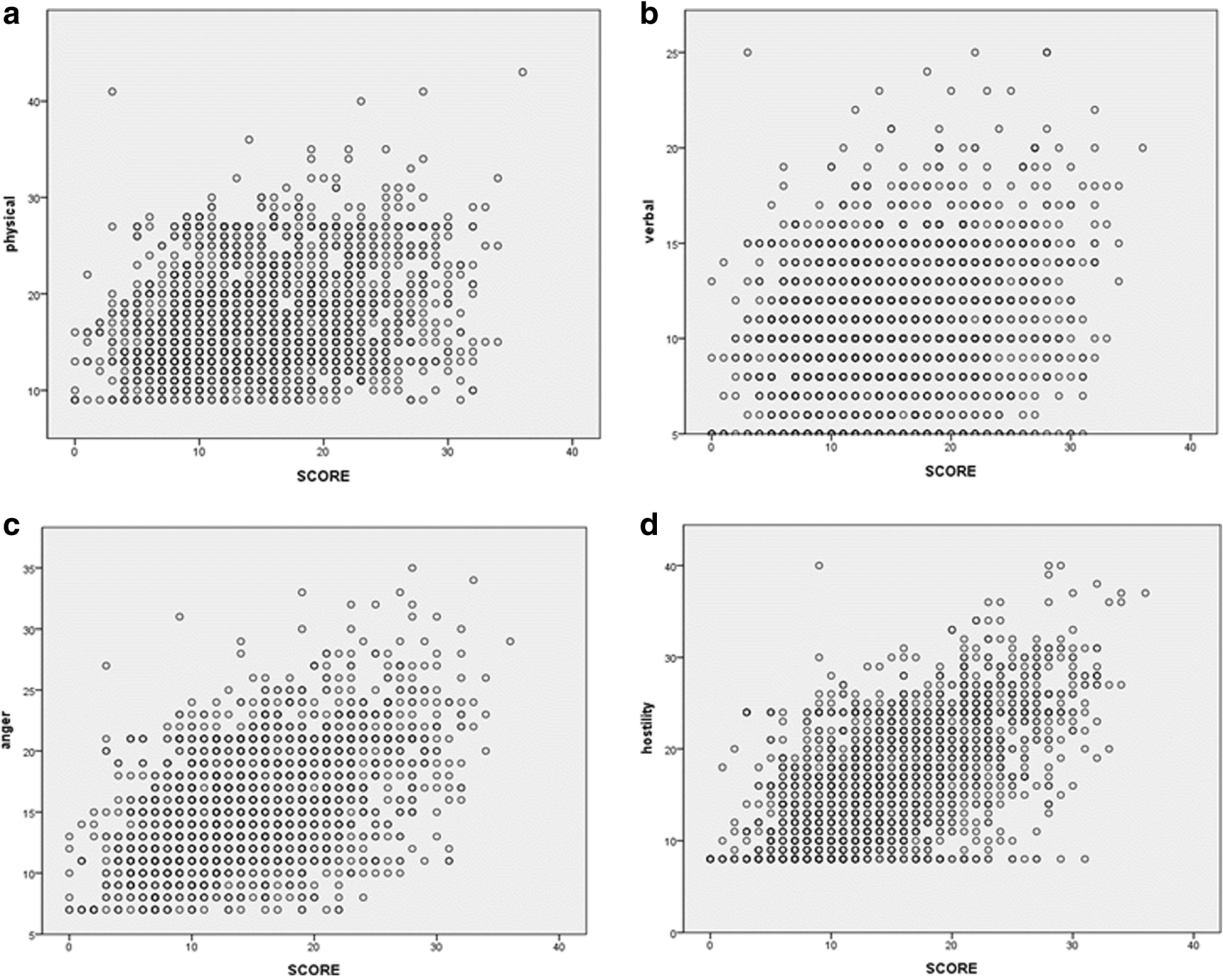
Correlations between RCMAS and aggressive propensity. **a** physical aggression, r = 0.272 **b** verbal aggression, r = 0.246 **c** anger, r = 0.501 and **d** hostility, r = 0.600

As the results of correlation analysis, aggression scores of anxiety group were higher than that of control group. Particularly, anger and hostility were more closely associated with anxiety than physical and verbal aggression in the subdomains of aggression. Pearson’s coefficients between anxiety and physical aggression, verbal aggression, anger, and hostility were 0.272, 0.246, 0.501 and 0.600, respectively (Fig. 1).

**Fig. 2:**
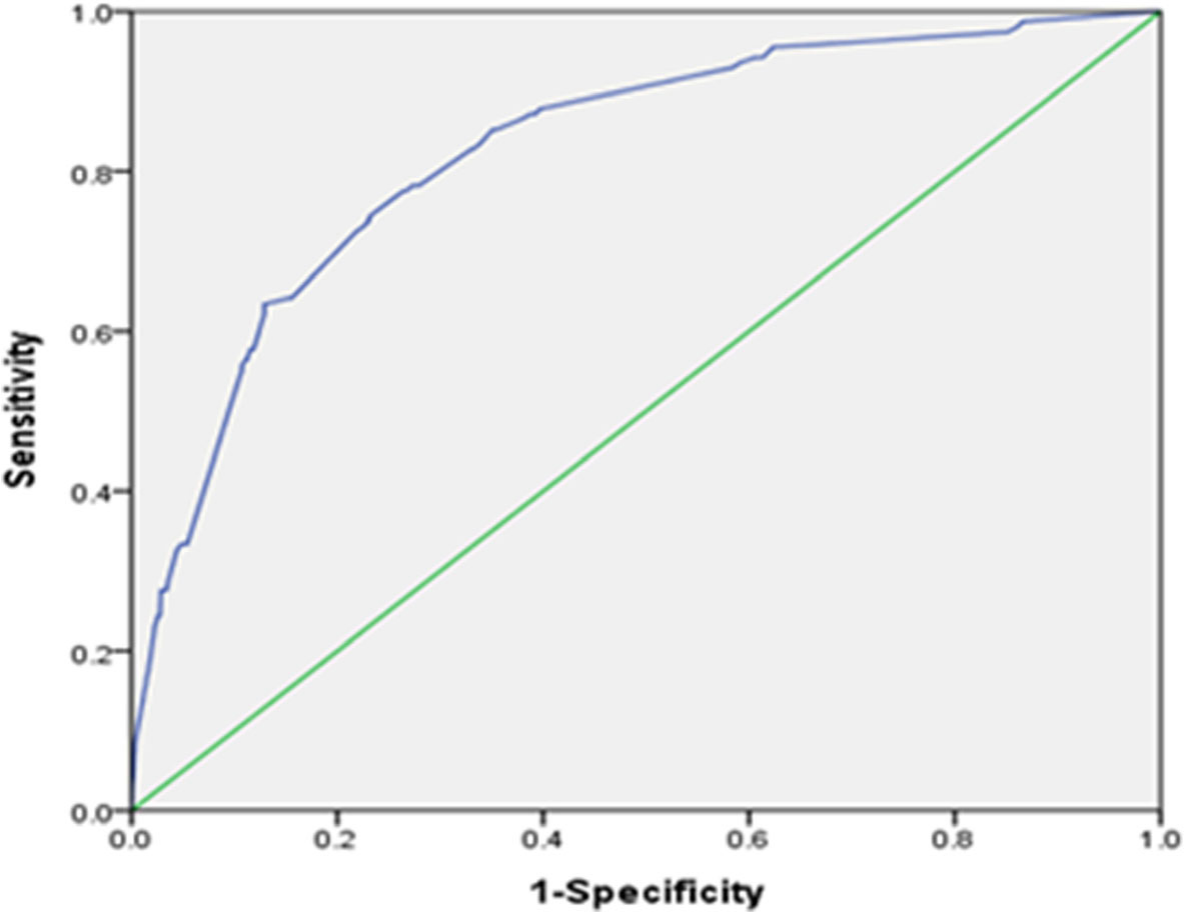
Area under receiver operating characteristic curve for aggressive propensity in anxiety group within model II that included sex, age, headache, scoliosis, constipation, asthma and total aggression (score ≥ 69) for analysis

To assess the risk of anxiety in relation to aggression score and to determine the cut-off score, two models were constructed in multivariate analysis. Model I included variables of sex (girls), age (under 15), medical conditions of headache, scoliosis, constipation, asthma and total aggression scales. Results revealed that the risk of anxiety significantly increased with asthma, headaches, female sex, age under 15, constipation, and total aggression score. Statistical analysis with AUROC revealed that total aggression score of 69 points had higher sensitivity (77.6%) and specificity (72.8%) to discriminate the probability of anxiety. The AUROC curve was 0.812 (95% CI = 0.777–0.848, *p* < 0.001). Therefore, in the model II, participants were divided into two groups using the cut-off of 69 points of aggression scores. The participants with an aggression score of 69 or higher had a nine-fold higher risk of anxiety than those under 69 (Table 3). In model II, the Hosmer-Lemeshow test revealed a good fit (χ2 = 2.592, *p* = 0.920) and AUROC was 0.828 (Fig. 2).

**Table 3:** Multivariate analysis for predictive factors of anxiety

Factors	Crude OR		Model IAdjusted OR	Model II Adjusted OR		
	estimate	95% CI	Estimate	95% CI	Estimate	95% CI
Girls	2.21^***^	1.56–3.14	2.19^***^	1.45–3.32	1.99^**^	1.33–2.96
≤15 yrs	1.59^**^	1.13–2.24	2.00^**^	1.30–3.06	1.89^**^	1.26–2.85
Headache	3.94^***^	2.81–5.54	2.32^***^	1.53–3.50	2.79^***^	1.88–4.15
Scoliosis	2.22^*^	1.30–3.50	1.80	0.95–3.40	1.90^*^	1.05–3.44
Constipation	2.27^***^	1.44–3.58	1.78^*^	1.02–3.10	1.84^*^	1.08–3.12
Asthma	2.62^*^	1.24–5.51	2.65^*^	1.11–6.33	2.50^*^	1.04–6.01
Total aggression scale	1.08^***^	1.06–1.09	1.08^***^	1.06–1.09		
Total (≥69)	9.28^***^	6.27–13.72			9.00^***^	6.00–13.51

^*^p < 0.05, ^**^p < 0.01, ^***^p < 0.001

Model I included variables of sex, age, caffeine, headache, muscle pain, scoliosis, constipation, indigestion, heartburn, asthma, pain reliever, digestant, sleeping pills and total aggression scale. Model II included all the variables as Model I except the total aggression scale. Instead, Model II used total aggression cut-off (score ≥ 69)

## Discussion

This study presents a clear and specific association between anxiety and aggression in Korean adolescents. In particular, among the subdomains of aggression, anger and hostility were more closely associated with anxiety than physical and verbal aggression; this indicates an interesting relationship between indirect aggression and anxiety.

Aggression often co-occurs with anxiety in childhood. Also, to some extent, adolescents may exhibit a combination of high aggression and anxiety [23]. Our results demonstrated higher total aggression score in the anxiety group than in controls. The AUROC was 0.812, indicating that the ability to predict anxiety is much better than by chance alone (0.5).

Especially, indirect aggression (anger and hostility) was more closely related with anxiety. Although many researchers have long recognized the significance of studying childhood aggression, only recently has attention been given to indirect forms of aggression. Unlike direct aggression, indirect aggression is an inconspicuous form of behavior that is difficult to detect. Therefore, teachers and parents are often unaware of who is indirectly aggressive, and therefore assessment of indirect aggression faces many complications. In this study, high level of indirect aggression was related with high level of anxiety, after adjusting confounders. As suggested in previous literature, being a victim of indirect aggression was associated with higher levels of mood disorders such as depression, loneliness, and anxiety. This was an understandable result considering that such an event could hurt standings in social groups, which is especially important in adolescence [24]. In a comparable context, the present study suggested that aggressive adolescents are at high risk of anxiety.

Adolescence is a period in which aggressive behavior tends to increase. Accordingly, it is essential to understand how the specific subtypes of aggression during adolescence contributes to anxiety increase, or vice versa. Previously, it was shown that aggression was a risk factor for the development of internalizing problems (depression and anxiety) in male adolescents. Although significant results were reported in that study, the relatively low internal consistencies associated with the anxiety measure likely weakened the reported results [4].

Girls are at a higher risk for anxiety than boys, revealing a girl-to-boy prevalence ratio of 2.3. This result is coherent with previous studies reporting sex differences in anxiety disorders. Varying influences from reproductive hormones and neurotransmitter expression were suggested to account for gender difference [25, 26].

In relation to medical conditions, students with headaches, asthma, scoliosis, and constipation were included in the high-risk group of anxiety. Numerous studies on pain comorbidity including headache have established an association between pain and psychiatric disorders [29]; in particular, this association is strongest for anxiety and depression [30]. The transition from childhood to adolescence is a sensitive and critical period for neurodevelopment. The developmental aspects of the nervous system may impact the advent of neurological disorders such as headaches. In addition, pediatric headache disorders are closely associated with negative psychological symptoms. Also, some previous studies revealed that anxiety and headache disorders have a significant correlation in girls [27, 28].

Our findings support previous research indicating that children with asthma demonstrate elevation of psychological difficulties [31]. In the case of scoliosis, it was reported that perception of spinal appearance was significantly associated with anxiety; accordingly, anxiety levels decreased after wearing back braces. Therefore, it may be assumed that scoliosis has a negative impact on mental health in adolescence, a period in which appearances and looks are extremely important [32]. Finally, constipation was also suggested as another risk factor for the anxiety group. Considering that the study population was consisted of a relatively young population without ample experience with life stressors, the aforementioned medical comorbidities may not be as manageable as for adults.

This study has an inherent limitation due to its cross-sectional study design and thus cannot lead to causal conclusions. Also, the survey was conducted in a specific region in Korea, rendering generalization difficult. However, the major strength of this study is the large number of subjects, providing sufficient statistical power. Also, it is the first study to assess the relationship between aggression and anxiety in Korean adolescents. Furthermore, the discovery of an aggression cut-off value that sorts out adolescents that are highly likely to be classified as the anxiety group will also aid further medical and/or psychological research.

## Conclusions

Our results demonstrated higher risk of anxiety with increasing total aggression score. In particular, indirect aggression (i.e. anger and hostility) was more closely associated with anxiety.

## Abbreviation

AOR: Adjusted odds ratio
AQ: Aggression Questionnaire
AUROC: Area under the receiver operator characteristics
CI: Confidence interval
OR: Odds ratio
RCMAS: Revised Children’s Manifest Anxiety Scale
